# Hepatitis B virus X protein and TGF-β: partners in the carcinogenic journey of hepatocellular carcinoma

**DOI:** 10.3389/fonc.2024.1407434

**Published:** 2024-06-19

**Authors:** Wei Yan, Dean Rao, Feimu Fan, Huifang Liang, Zunyi Zhang, Hanhua Dong

**Affiliations:** ^1^ Hepatic Surgery Center, Tongji Hospital, Tongji Medical College, Huazhong University of Science and Technology, Wuhan, Hubei, China; ^2^ Hubei Key Laboratory of Hepato-Pancreato-Biliary Diseases, Wuhan, Hubei, China; ^3^ Hubei Province for the Clinical Medicine Research Center of Hepatic Surgery, Wuhan, Hubei, China; ^4^ Key Laboratory of Organ Transplantation, Ministry of Education, National Health Commission (NHC), Chinese Academy of Medical Sciences, Wuhan, China

**Keywords:** HBx protein, hepatitis B virus, TGF-β signaling, hepatocellular carcinoma, tumor suppressor, pro-tumorigenic

## Abstract

Hepatitis B infection is substantially associated with the development of liver cancer globally, with the prevalence of hepatocellular carcinoma (HCC) cases exceeding 50%. Hepatitis B virus (HBV) encodes the Hepatitis B virus X (HBx) protein, a pleiotropic regulatory protein necessary for the transcription of the HBV covalently closed circular DNA (cccDNA) microchromosome. In previous studies, HBV-associated HCC was revealed to be affected by HBx in multiple signaling pathways, resulting in genetic mutations and epigenetic modifications in proto-oncogenes and tumor suppressor genes. In addition, transforming growth factor-β (TGF-β) has dichotomous potentials at various phases of malignancy as it is a crucial signaling pathway that regulates multiple cellular and physiological processes. In early HCC, TGF-β has a significant antitumor effect, whereas in advanced HCC, it promotes malignant progression. TGF-β interacts with the HBx protein in HCC, regulating the pathogenesis of HCC. This review summarizes the respective and combined functions of HBx and TGB-β in HCC occurrence and development.

## 1 Introduction

Liver cancer is a substantial health problem globally, and it is estimated to be the 6^th^ most frequent tumor and the 3^rd^ primary reason for cancer mortalities (1). In 2020, approximately 905,700 and 830,200 people were diagnosed and died, respectively, due to this tumor worldwide. Between 2020 and 2040, a 55.0% annual increase in new liver cancer diagnoses is anticipated, with 1.4 million new cases expected (2). Various factors contribute to liver cancer development, including alcohol, metabolic syndrome, type 2 diabetes, obesity, non-alcoholic fatty liver disease (NAFLD), aflatoxin B1, and tobacco (3, 4). However, HBV is still the most significant cause as it leads to 56% of the cases, according to GLOBOCAN (5). The most common type of liver cancer, HCC, accounts for nearly 75% of all cases (6)^(pp1978–2012)^.

**Figure 1:**
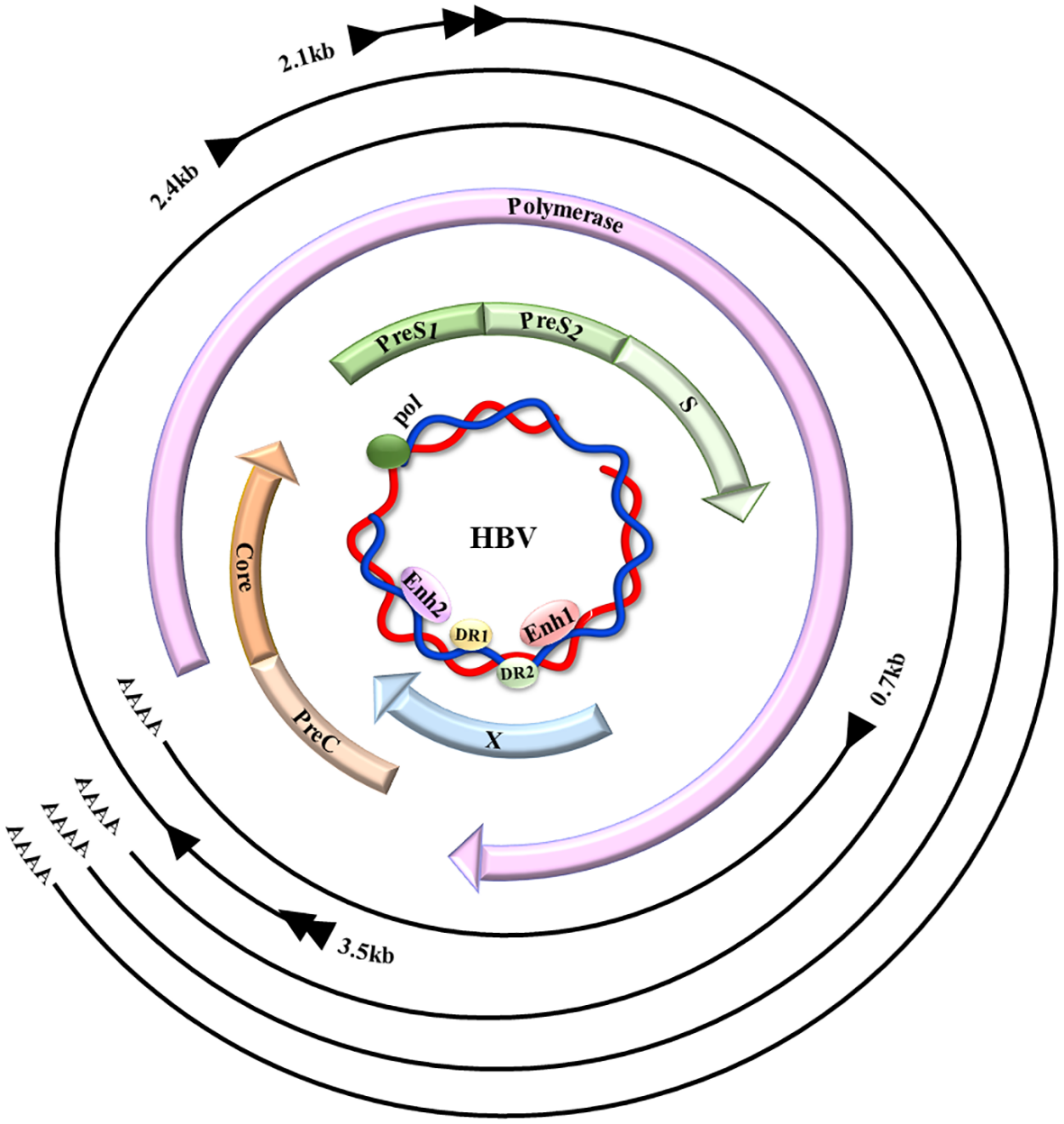
HBV genome map. HBV comprises a small, partially ~ dsDNA genome (the inner blue circle), which contains four promoters and two enhancer regions (Enh1/2), in addition to two direct repeats (DR1/2). The four HBV-coded overlapping ORFs: preS/S, precore/C, polymerase, and X, are indicated by the colored arrows. When the virus is replicating, the rcDNA enters the nucleus, it undergoes conversion into cccDNA through the action of host DNA polymerase and repair enzymes, that act as the viral transcription template, showing the primary HBV transcripts (outer black lines), their 5′ initiation sites (black arrowheads), aside with 3′ poly-A tails (AAAA).(This figure is modified from **Figure 1** of the article PMID: 35648301) (11).

HBV, a non-cytopathic DNA virus from the Hepadnaviridae family, causes diseases of liver when transmitted through infected blood or body fluids (7). The viral genomes contain relaxed circular DNA (rcDNA), which encodes numerous proteins and can transform into covalently closed circular DNA (cccDNA) in the nucleus (8–10) (**Figure 1**).

The HBx protein, comprising 154 amino acids and a 17 kDa molecular weight, and the variation in amino acid sequence of HBx protein is more pronounced between different HBV genotypes than within the same HBV genotype (12). It is named after its encoding gene due to unknown homologous proteins (13). HBx is recruited to cccDNA microchromosomes and enhances their transcription as HBV replicates; alongside being necessary for HBV cccDNA transcription/viral replication (14, 15), it is assumed to be involved in hepatocarcinogenesis. HBx is primarily found in the cytoplasm of hepatocytes, with some residing in the nucleus. HBx activates transcription by interacting with nuclear transcription factors (TFs) while indirectly bound to DNA. Additionally, HBx functions as an adaptor or kinase activator that influences signal transduction pathways (16, 17).

Transforming growth factor-β (TGF-β) is a pleiotropic cytokine involved in numerous physiological and pathological processes, including tumorigenesis (18–21). It performs contradictory roles in early and advanced liver cancer, inhibiting cell proliferation and inducing apoptosis in early-stage lesions while promoting cancer progression in advanced-stage HCC via fibrosis, invasion, and epithelial-mesenchymal transition (EMT) (22, 23). TGF-β signaling involves Samd-dependent and -independent pathways (21, 24).

As stated, TGF-β and HBx are expressed abnormally in HCC and contribute to its development and progression. HBx expression was positively correlated to TGF-β activity in hepatocytes of cirrhotic/cancerous/chronic hepatitis patients. The results suggested that HBx expression might induce TGF-β1 expression in early-stage HBV infection (25). Additionally, HBx regulates the transition of tumor-suppressive pSmad3C signaling to oncogenic pSmad3L signaling (26), deeming necessary evidence that HBx is intensely contributing to TGF-β carcinogenic effect in HCC.

In the following section, we will examine the specific mechanisms of action of HBx and TGF-β in the progression of HBV- associated HCC and their complex functional cross-talk and interactions.

## 2 Oncogenic mechanism of HBX

**Figure 2:**
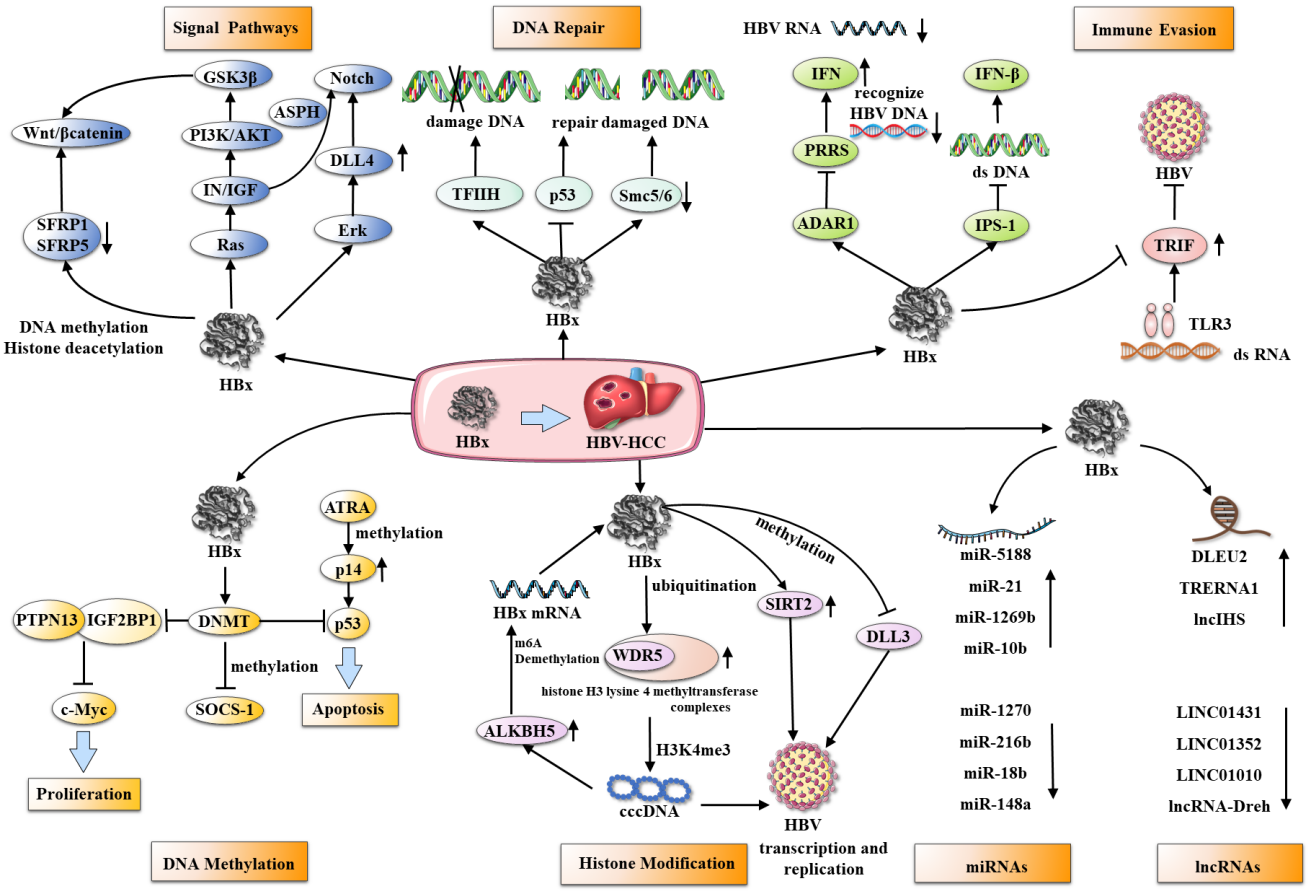
HBx and its multifunctional roles in hepatocarcinogenesis. HBx interacts with several cellular targets through various mechanisms, including affecting multiple signaling pathways, damaged DNA repair, immune evasion, and epigenetic changes (DNA methylation, histone acetylation, ncRNAs) to accelerate HBV transcription, replication, and malignant progression of HCC.

HBx contributes to HCC development and progression by regulating multiple pathways, epigenetic changes, gene expression, and transcription. (**Table 1**, **Figure 2**).

**Table 1:** A summary of the mechanisms by which HBx leads to HCC.

Type	Sub-Type	Target	Mechanism	Reference
Pathways	Proliferation and invasion	Wnt/β-catenin	HBx induces EMT and proliferation by activating the Wnt/β-catenin signaling pathway	(27, 28)
		Notch	Notch-1, Jagge-1 and HECA-1 are upregulated by HBx to promote cancer	(29, 30)
		IN/IGF	HBx activates IN/IGF pathway to enhance HCC cell metastasis, migration, and invasion	(31)
	DNA Repair	p53	p53-mediated repair of damaged DNA is inhibited	(32)
		TFIIH	The interaction between HBx and TFIIH blocks DNA repair	(33)
		Smc5/6	Hbx-induced Smc5/6 degradation inhibits HR repair	(34)
	Immune Evasion	TRIF	HBx protein can down-regulate TRIF to induce immune escape	(35)
		IPS-1	HBx interacts with IPS-1 to inhibit the activation of IPS-1	(36)
		ADAR1	HBx promotes ADAR1 expression to enhance immune evasion	(37)
Epigenetics	DNA Methylation	DNMT1	HBx enhances regional hypermethylation of tumor suppressor genes and inhibits the transcription of PTPN13 by upregulating DNMT1	(38–40)
		DNMT3A/3B	HBx-increased DNMT3A/3B enhances CpG island methylation in the SoCS-1 promoter, and it antagonizes ATRA-mediated p53-dependent apoptotic pathways	(41, 42)
	Histone Modification	WDR5	HBx enhances HBV transcription by promoting H3K4me3 modification through upregulation of WDR5	(43)
		SETDB1	HBX inhibits SETDB1-mediated HBV suppression	(44)
		SIRT2	HBX upregulates SIRT2 to promote HBV transcription and replication	(45)
		DLL3	HBx prevents cancer cell apoptosis through histone acetylation leading to the silencing of DLL3	(46)
	miRNAs	miR5188	HBx-induced miR5188-FOXO1/β-catenin-c-Jun feedback loop promotes HCC stemness, metastasis, proliferation, and chemoresistance	(47)
		miR-21	HBx inhibits the expression of the tumor suppressor gene PDCD4 by inducing overexpression of miR-21	(48)
		miR1269b	HBx upregulates miR1269b in an NF-κB signal-dependent manner, targeting and increasing CDC40 to promote HCC progression	(49)
		miR-106b	HBx leads to increased transcription of miR-106b to promote HCC	(50)
		miR1270	HBx can upregulate CENPM through miR- 1270 downregulation, which promotes hepatocarcinogenesis	(51)
		miR-216b	HBx can down-regulate cancer-suppressing miR-216b to promote HCC	(52)
		miR-122	HBx-LINE1 promotes cell migration by depleting miR-122	(53)
		miR-18b	HBx inhibits the expression of miR-18b by upregulating NUSAP1, thus promoting cancer	(54)
		miR-148a	HBx inhibited the p53-induced activation of miR-148a and reversed the inhibitory effect of miR-148a on the HPIP/mTOR pathway	(55)
	lncRNAs	DLEU2	HBx and DLEU2 co-recruit on cccDNA and regulate its transcription	(56)
		LINC01431	HBx-LINC01431-PRMT1 feedback loop facilitates HBV replication and immune evasion	(57)
		TRERNA1	TRERNA1 upregulated by HBx triggers the RAS/Raf/MEK/ERK pathway and increases resistance to sorafenib	(58)
		LINC01010	HBx promotes the progression of HCC by down-regulating the expression of LINC01010	(59)
		lncRNA-Dreh	HBx reduces the expression of lncRNA-Dreh to promote the development of HCC	(60)
		lncIHS	HBx-induced lncIHS expression to activate AKT/GSK-3b and ERK pathways	(61)
HBx mutation	Ct-HBx	TXNIP	Ct-HBx transactivates NFATC2 to transcriptionally repress TXNIP, thus promoting the HCC	(62)
		C-jun/AP1	Ct-HBx enhances the invasion and metastasis of HCC cells through C-Jun/AP1 signal activation	(63)
		MMP10	Ct-HBx promote the HCC’s invasion and metastasis by increasing MMP10	(63)
		Cav1	Ct-HBx activates the Cav1/LRP6/β-catenin/FRMD5 axis to enhance hepatocarcinogenesis	(64)
		FXR	Anticancer FXR signal is weakly co-activated by Ct-HBx compared to full-length HBx	(65)
	N-terminal mutation	F30V	F30V enhanced the phosphorylation of PI3K-Akt, thereby enhancing the antiapoptotic activity of HBx, and it affects the binding of HBx to cccDNA to promote immune evasion	(66)

### 2.1 HBx regulatory signaling pathway leads to HCC

HBx is a key player in the etiology of HCC and manipulates various biochemical pathways within host cells, which are vital for physiological and biochemical functions. HBx redirects these pathways to promote cell proliferation and invasion, impair DNA repair, and evade immune responses, collectively contributing to tumorigenesis.

In particular, the Wnt/β-catenin, Notch signaling pathways, and the insulin-like growth factor (IN/IGF) pathway have been associated with the organization of HBx’s carcinogenic influence. The Wnt/β-catenin pathway is upregulated in over 90% of HCC cases (67), it is activated via HBx ectopic expression combined with Wnt-1, nevertheless, this activation is crucial for stabilizing the effect of HBx on β-catenin (27). In turn, the inhibition of Wnt/β-catenin pathway antagonists, SFRP1 and SFRP5, by HBx increases HCC cell proliferation and epithelial-mesenchymal transition (EMT) (28). Concurrently, HBx also leads to prolonged activation of the oncogenic pathway Notch by upregulating its receptors and ligands, particularly Notch-1 and Jagged-1 (29, 30). This Notch pathway activation is further amplified by HBx’s effect on the Erk (MEK1/2) and PI3K/AKT pathways (68). Furthermore, HBx alters the IN/IGF pathway, which acts in tandem with the Wnt/β-catenin pathway. IN a double-transgene mouse model of HBx and IRS1 that mimic human hepatocellular carcinoma, persistent activation and cross-talk of IN/IGF1, WNT/β-catenin, and Notch enhance HCC cell metastasis and invasiveness, and up-regulation of aspartate β-hydroxylase (ASPH) is central to these signaling cascades (31). The above mechanism exploration is primarily based on mouse models and cells; however, further clinical experiments are required to validate its potential as a therapeutic target for liver cancer patients.

HBx also compromises DNA repair, a critical tumorigenic facet of HBV-induced HCC. The HBx protein can impede the repair of damaged DNA mediated by the anti-oncogene p53 (32), inhibition of transcription factor IIH (TFIIH) (33), and degradation of Smc5/6 complex (34). This disruption of DNA repair processes leads to the accumulation of DNA damage, subsequently instigating the onset of hepatocarcinogenesis.

On the immune evasion front, HBx uses several strategies to avoid the innate immune response, particularly suppression of interferons. It suppresses Toll-like receptor 3 (TLR3) and its adaptor protein, TIR-domain containing adaptor protein inducing interferon-β (TRIF), thereby reducing the anti-HBV immune responsee (35). Furthermore, HBx inhibits interferon β (IFN-β) signaling by interacting with IPS-1, suppressing interferon induction during HBV infection (36). Moreover, HBx facilitates adenosine deaminases acting on RNA 1 (ADAR1)-mediated viral RNA editing, which prevents the recognition of HBV RNA by pattern recognition receptors, thereby inhibiting interferon production (37). Patients with HBV immune tolerance experience a higher incidence of HCC compared to those who receive immune activation therapy (69). Type I interferons are approved as a first-line treatment for chronic HBV. However, it is speculated that the effectiveness of IFN therapy may be limited due to inhibition by HBx, and its significant side effects limit its broader clinical application. Thus, there is a need to develop more effective immunotherapeutic drugs to curb the progression of HBV-associated HCC.

### 2.2 HBx causes HCC through epigenetic mechanisms

In the context of HBV or HCV infection, HCC development and progression are supported by epigenetic dynamics. This is associated with DNA methylation, histone modifications, microRNAs (miRNAs), and long noncoding RNAs (lncRNAs).

In liver tissue affected by chronic hepatitis or cirrhosis due to HBV or HCV infection, DNA methylation can be detected, which is considered a precancerous state of HCC (70). Significantly elevated HBx expression is associated with methylation abnormalities in HBV-infected HCC patients (38). DNMT1, responsible for maintaining DNA methylation during replication, and DNMT3A/3B, also known as *de novo* methyltransferases, are involved in methylation modifications (11). HBx upregulates DNMT1/3A1/3A2, resulting in the hypermethylation of tumor suppressor-related genes (38, 39), such as increasing CpG island methylation of the cytokine signaling-1 (SOCS-1) promoter (41) and hypermethylation of p14’s promoter (42). HBx also inhibits Tyrosine-protein phosphatase nonreceptor type 13 (PTPN13) transcription by upregulating DNMT3A and promoting DNA methylation, thereby facilitating the progression of HCC progression (40).

Epigenetic modification of histones contributes to multiple malignant tumor pathogeneses and metastases, including HCC (71–73). HBx inhibits H3 lysine 4-methyltransferase complex core subunit WDR5 ubiquitination, resulting in increased HBV transcription via H3K4me3 modification of cccDNA (43). A positive feedback loop is formed when the HBx-WDR5-H3K4me3 axis increases ALKBH5, a demethylase enzyme that catalyzes m6A demethylation of HBx mRNA (74). However, other reports indicate that H3K4me3 is generally diminished in HBV infections. HBx can alleviate the reduction in H3 acetylation and H3K4me3, as well as the methylation of histone 3 lysine 9 (H3K9me), following HBV infection. This action helps to mitigate the transcriptional silencing of cccDNA (45). HBx inhibits cell apoptosis by inducing DLL3 silencing via histone acetylation in HBV-associated HCC (46).

MicroRNAs (miRNAs) and long noncoding RNAs (lncRNAs) are two important classes of noncoding RNAs that substantially impact cellular processes, including differentiation, proliferation, and survival (75, 76).Notably, irregular expressions of these RNA types are characteristic of liver disorders, including HCC (77, 78). HBx is a key mediator in hepatocarcinogenesis, influencing the levels of both miRNAs and lncRNAs to promote or inhibit HCC development (79, 80). For example, HBx can induce the expression of miR-5188 through Wnt signaling, thereby establishing a feedback loop that promotes HCC stemness, metastasis, and chemoresistance (47). Additionally, it can modulate the expression of miR-21, miR-1269b, and miR-106b, thereby affecting tumor suppression and the progression of HCC (48–50, 81)^(p12)^. In contrast, downregulated miRNAs affected by HBx, such as miR-1270, miR-216b, miR-122, miR-18b, and miR-148a, are involved in HCC carcinogenesis via numerous pathways (51–55). HBx influences transcription, translation, and epigenetic regulation of lncRNAs, with implications for HBV replication, immune evasion, drug resistance, and the activation of numerous signaling pathways (80). Noteworthy examples include DLEU2, LINC01431, TRERNA1, LINC01010, lncRNA-Dreh, and lncIHS (56, 57) ^(p1)^, (58–61). Consequently, these interactions between HBx and noncoding RNAs emphasize their significance in the molecular mechanisms underlying the development and progression of HBV-associated HCC.

### 2.3 HBx mutation leads to HCC progression

HBV DNA integration into the host genome induces genomic instability and direct insertion mutations in cancer-related genes in early-stage clonal HCC expansion (82). Variation in the HBx sequence, specifically at the 3’-terminus, substantially affects the HCC development. Chimeric transcripts of HBx and human genes, often including 3’-terminal deletions, are frequently expressed and can encode alternative HBx versions implicated in transcriptional regulation (83, 84).

The carboxyl terminus of HBx plays multiple roles in protein-protein interactions, transcriptional transactivation, DNA repair, cellular signaling, and HCC pathogenesis (85). COOH-terminal mutations are associated with HCC and may lead to reactive oxygen species (ROS) production, which damages mitochondrial DNA (86, 87). The clustering pattern of HBx 3’ end generates a truncated X protein (Ct-HBx) (62) that promotes HCC progression by mediating glucose metabolism reprogramming, increasing matrix metalloproteinase 10 (MMP10) transcription (62, 63), and enhancing Wnt/β-Catenin signaling (64). HBX-C30 (30 aa deletion from HBx C terminus) co-activates anticancer FXR signaling less effectively than full-length HBx (65). Additionally, mutations in the N-terminal domain, such as F30V, increase HBx’s antiapoptotic activity and diminish HBV’s replication efficacy. These HBx mutations facilitate the development of HCC and immune evasion (66).

### 2.4 Therapeutic approach for HBx

Currently, there is a lack of drugs and therapeutic methods targeting HBx, which awaits further development by researchers. During HBV infection, the host SMC complex, SMC5/6, suppresses viral transcription. HBx promotes ubiquitination and degradation of SMC5/6, enhancing cccDNA transcription and increasing viral replication. This destabilization of SMC5/6 aids HBV in evading host immune surveillance and facilitates viral persistence and propagation (15, 88, 89). Nitazoxanide, an FDA-approved thiazolide antimicrobial for protozoal enteritis, and Pevonedistat, an NAE inhibitor approved for myelodysplastic syndromes, have demonstrated *in vitro* efficacy in inhibiting the HBx/SMC5/6 axis. This inhibition restores SMC5/6 protein levels, effectively suppressing HBV transcription and translation (90–92). Dicoumarol, the precursor to warfarin and a competitive NQO1 inhibitor, reduces HBx level and cccDNA transcription in both HBV-infected hepatocytes and humanized mouse models (93).

## 3 TGF-β

### 3.1 Overview of the TGF-β Signaling

The TGF-β branch, classified as the TGF-β family, is activated by three ligands: TGF-β1-3, with TGF-β1 being the most abundant and typical isomer as it is secreted by almost all cells (94). The TGF-β ligand synthesis is in a longer precursor protein that is cleaved by furin protease; additionally, TGF-β disulfide-bonded dimers and the latency-associated peptide (LAP) are joined by non-covalent bonds to form a small latent complex, which is cross-linked with the latent TGF-β binding protein (LTBP) forming a large latent complex. This inactive complex is then secreted and associated with the extracellular matrix (ECM) (95). Activation occurs when particular signals initiate the release of active TGF-β from the complex, involving elements such as extreme pH, proteases, and integrins (96). Upon TGF-β ligand binding, TβRII dimerizes and recruits TβRI to produce a heterotetrameric TβRI-TβRII complex (97, 98).

**Figure 3:**
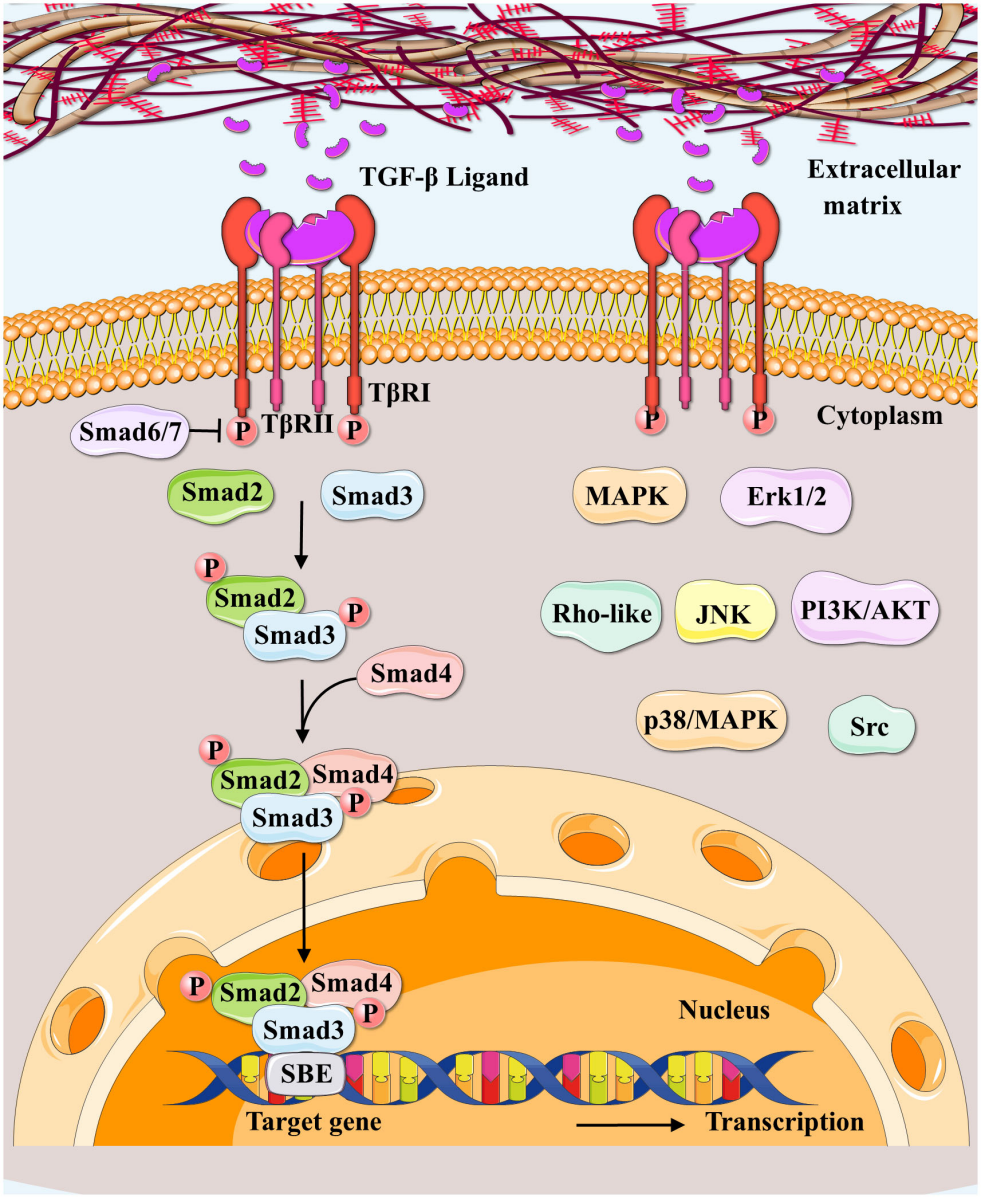
Canonical and non-canonical TGF-β signaling pathways. Regarding canonical TGF-β signaling pathways (SMAD‐dependent signaling pathways), the TGF-β ligand secreted by extracellular matrix binds to TGFβRII to initiate this pathway; and TGFβRII upon activation, forms a complex with TGFβRI and phosphorylates TGFβRI. Then Smad2/3/4 forms transcription complexes, entering the nucleus besides binding to DNA to regulate the target gene expressions. Smad6/7 are canonical TGF-β pathway inhibitors. Non-canonical Smad pathways (SMAD‐independent signaling pathways) include MAPK, Erk1/2, Rho-like, PI3K/AKT, JNK, p38/MAPK, and Src tyrosine kinase pathways.

TβRI phosphorylates R-Smads (Smad2/3) at their extreme C-terminal Ser-X-Ser motifs in the canonical Smad signaling, resulting in oligomerization with Co-Smad (Smad4) and nuclear translocation (99). The activated Smad4-R-SMAD complex regulates the transcription of target genes through interactions with DNA-binding transcription factors (20). The Smad linker can also be phosphorylated to activate numerous signaling pathways involved in pathological or pathophysiological gene expressions (100). Inhibitory Smad6/7 (I-Smads) modulate canonical Smad signaling in a feedback manner via various mechanisms (101, 102). Besides canonical Smad signaling, TGF-β functions by activating non-canonical Smad pathways, including the mitogen-activated protein kinase (MAPK), extracellular signal-regulated kinases1/2 (Erk1/2), Rho-like, phosphatidylinositol-3-kinase (PI3K)/AKT, c-Jun amino-terminal kinase (JNK), p38/MAPK (103), and the Src tyrosine kinase signaling pathways (104, 105) (**Figure 3**).

### 3.2 TGF-β Signaling in HCC

**Figure 4:**
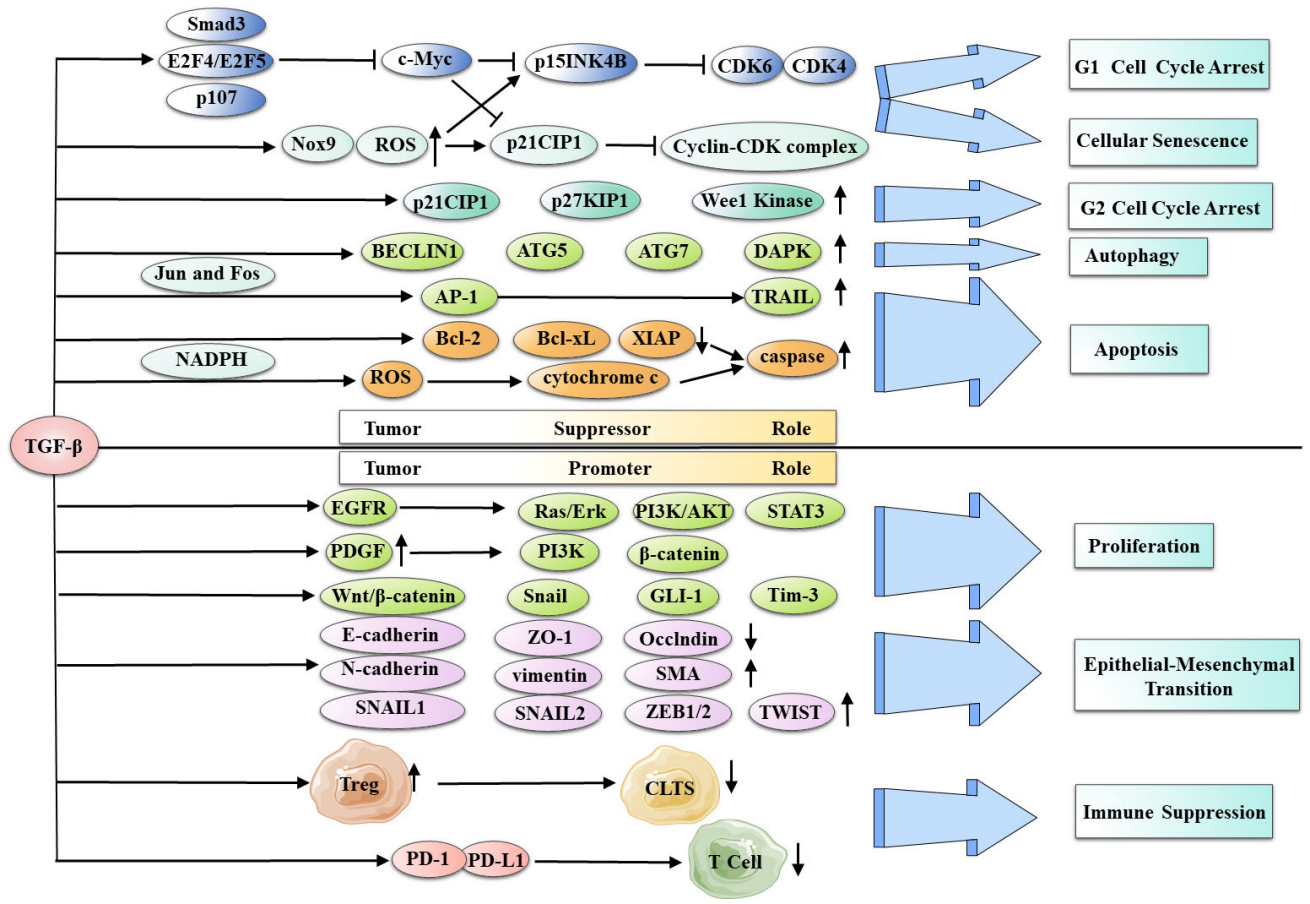
TGF-β dichotomous role in HCC development and progression. TGF-β has various tumor-suppressing functions, including G1 and G2 cell cycle arrest, cellular senescence, autophagy, and apoptosis. Conversely, it has the ability to serve as a tumor promoter, inducing cancer cell proliferation, EMT, and immune suppression in HCC.

TGF-β signaling is essential at every stage of liver disease progression, from initial inflammation and damage to fibrosis, cirrhosis, and ultimately liver cancer. Early on, it inhibits tumor growth by inducing senescence and apoptosis. It promotes tumor growth, EMT, and metastasis in advanced stages. Nevertheless, Smad-dependent and independent pathways are involved in the complex signaling (106). (**Figure 4**).

#### 3.2.1 Tumor suppressor roles of TGF-β in HCC

##### 3.2.1.1 Cell cycle arrest

TGF-β induces HCC cell cycle arrest by suppressing transcriptional factor expression, including the pro-growth TF c-Myc, which is mediated through nuclear translocation of a complex consisting of Smad3, E2F4, or E2F5, and RB-related factor p107 (107, 108). It also regulates cyclin-dependent kinases (CDKs) and their inhibitors, leading to G1 arrest (109). CDK2 binds to cyclin E to drive the cell cycle, while CDK4 or CDK6 binds to cyclin D (110). Furthermore, TGF-β reduces the inhibitory effects of c-Myc on CDK inhibitors p21CIP1 and p15INK4B, leading to cell cycle arrest (111–113)^(p1)^, (114). The accumulation of NADPH oxidase-4 (Nox4) and ROS in well-differentiated HCC cell lines further enhances the expression of TGF-β-induced p21CIP1 and p15INK4B (115). In addition to G1 phase arrest, TGF-β causes HCC-cell G2 phase arrest by inducing CDK inhibitors p21CIP1 and p27KIP1, along with Wee1 kinase (116)^(p1)^.

##### 3.2.1.2 Cellular senescence

Cellular senescence is a persistent cell cycle arrest state inhibiting tumor progression (117–119). There is a significant relationship between tumor inhibition and cell senescence in HCC. Telomerase and telomeres are mainly involved in malignant cell senescence and hepatocyte aging (120), with telomerase reactivation promoting uncontrolled proliferation and malignant transformation in HCC (121). In malignant tumors, TGF-β/Smad signaling inhibits human telomerase reverse transcriptase (hTERT), demonstrating a regulatory role for TGF-β in HCC cell senescence (122–124).

##### 3.2.1.3 Autophagy

Autophagy is responsible for the degradation of proteins and cells (125, 126). Through Smad and non-Smad pathways, TGF-β induces autophagy in HCC cells by upregulating the expression of autophagy-related genes, including BECLIN1, ATG5, ATG7, and DAPK. Autophagy is closely related to TGF-β-mediated growth inhibition of HCC cells. Autophagy gene knockdown reduces TGF-β-mediated growth suppression and decreases pro-apoptotic gene expression. HCC cells are more resistant to TGF-β-induced autophagy than breast cancer cells, indicating its significance in growth suppression (127, 128). However, recent studies have found that tumor cells in HCC rely on autophagy for survival. This process can promote tumor development by inducing autophagic cell death in liver (129, 130). Therefore, the role of TGF-β-induced autophagy in liver cancer requires further research to clarify.

##### 3.2.1.4 Apoptosis

In liver cells, TGF-β induces apoptosis and inhibits cell proliferation (131). It initiates apoptosis through the death receptor pathway by mediating the activation of the TNF-related apoptosis-inducing ligand (TRAIL) promoter’s AP-1 site via Jun, Fos, and Smad proteins (132)^(p1)^, (133). TGF-β can induce apoptosis in liver cancer cells via the mitochondrial and death receptor pathways. TGF-β can also induce apoptosis via the mitochondrial pathway by reducing the expression of antiapoptotic B-cell lymphoma 2 (Bcl-2) family proteins and activating caspases (134). ROS generation via NADPH oxidase is essential for TGF-β-induced HCC cell apoptosis (135, 136). The apoptotic response can be suppressed by the epidermal growth factor receptor (EGFR) pathway (137, 138).

#### 3.2.2 Tumor promoter role of TGF-β in HCC

##### 3.2.2.1 Cancer cell proliferation

Most malignant tumors exhibit an increase in cell proliferation. TGF- promotes HCC proliferation via several pathways. Through the EGFR, TGF-β activates Ras/Erk, PI3K/AKT, and STAT3 signaling, resulting in hepatocyte proliferation. In addition, it upregulates the expression of PDGF to activate the PI3K and β-catenin pathways. TGF-β also activates other pathways, such as Wnt/β-catenin, Snail, and GLI-1, contributing to cancer cell proliferation (139–143). Furthermore, TGF-β/SMAD2 signaling induces c-KIT receptor ligand (stem cell factor) expression, activating c-KIT/JAK1/STAT3 signaling and establishing a positive feedback loop for HCC proliferation (139). The p38 and PI3K/AKT pathways mediate the PRL-3-induced TGF-β1 by activating FAK and developing another positive feedback loop (144).Thus, TGF-β interacts with multiple carcinogenic pathways in tumors, and blocking TGF-β activation could serve as a vital approach in the treatment of liver cancer.

##### 3.2.2.2 Genetic alteration and epigenetic modification

HCC development involves genetic, epigenetic, and transcriptomic mechanisms. Approximately 40% of HCC cases contain mutations in TGF-β pathway genes. Overexpression of TGF-β pathway genes is associated with inflammation and fibrosis, whereas their down-regulation inhibits tumor growth (145). Several TGF-β target genes implicated in HCC are overexpressed, including VEGFA, COL4A1, SNAI2, DAPK2/3, CDKN1A, and CDKN1. Genomic instability in HCC is caused by mutations in the TGF-β pathway and its non-canonical targets, including the JNK/MAPK/IKK, ERK/MAPK, RHO-ROCK, and PI3K/AKT pathways (146).

Epigenetic modifications increase the carcinogenic influence of TGF-β on HCC. In early HCC, demethylation of the Smad4’s promoter suppresses tumor growth, whereas hypomethylation of Smad7 and SNAI1 in the promoter region facilitates EMT recurrence and metastasis, it has been reported that the use of decitabine may drive liver cancer progression towards a pro-carcinogenic direction through this pathway (147). Methylation of the TTP promoter eliminates the post-transcriptional regulatory function of c-Myc, shifting TGF-β signaling from proliferation inhibition to promotion (148, 149). In HCC, high methylation levels frequently lead to the inactivation of the tumor suppressors RUNX3 and Smad Interacting Protein-1 (SIP1), which interact with Smads (150, 151). In conjunction with HDACs and G9 methyltransferase, the TGF-β-induced overexpression of SNAIL2 suppresses E-cadherin and enhances the invasiveness and metastasis of HCC cells (152).

##### 3.2.2.3 Epithelial-mesenchymal transition

EMT triggers epithelial cells to acquire mesenchymal properties, leading to migration, invasion, stemness, and resistance to apoptosis and immune responses (153, 154). TGF-β plays a fundamental role in triggering EMT in HCC (154, 155)^(p1)^. It downregulates epithelial markers (E-cadherin, ZO-1, and Occludin) and upregulates mesenchymal markers (N-cadherin, vimentin, and SMA) via Smad and non-Smad pathways (Rhogtase, MAPK, and PI3K/AKT/mTOR) (156, 157). TGF-β induces EMT-TFs, including SNAIL1/2, ZEB1/2, and TWIST. Smads interact with Notch, Hedgehog, Wnt, and Hippo signaling pathways to reprogram EMT-related genes. Additionally, miRNAs (158), such as the SNAIL-miR-34 and ZEB1-miR-200 feedback loops, regulate EMT during TGF-β stimulation (159). Long noncoding RNA also activated by TGF-β (lncRNA ATB) promotes ZEB1/2 overexpression via competitive binding with the miR-200 family, thereby inducing EMT and invasion (160).

##### 3.2.2.4 Immune suppression

TGF-β, by modulating immune cells involved in immune homeostasis and tolerance, acts as a critical inhibitor of both adaptive and innate immunity. This creates an immunosuppressive tumor microenvironment that facilitates tumor progression (161). Induction and differentiation of liver Treg cells by TGF-β contribute to immunosuppression (162) via numerous cell types, such as LSECs, HSCs, CAFs, TAMs, and MDSCs. Treg cells suppress immune responses, enhance cell proliferation, and deplete cytotoxic T lymphocytes (CTLs).TGF-β upregulates PD-1 and PD-L1, inhibiting TCR signaling and T cell proliferation and leading to T cell depletion in HCC (146). In addition to inhibition of T cells, TGF-β increases alternative macrophage activation by enhancing Tim-3 transcription in tumor-associated macrophages (TAMs), leading to the growth of HCC (163).

### 3.3 Therapeutic approach for TGF-β in HCC

The TGF-β pathway plays a crucial role in the progression of HBV-associated HCC. Many drugs targeting the TGF-β pathway have shown promising results in treating HCC (164). However, the clinical applicability and efficacy of these treatments require further validation through extensive clinical trials. Studies have shown that various chemotherapeutic agents, including Fluorofenidone (AKF-PD) (165), Sanguinarine (San) (166), Aspirin (167), Praziquantel (PZQ) (168), and Ursodeoxycholic acid (UDCA) (169), along with small molecule inhibitors such as Galunisertib (LY2157299) (170–172), LY2109761 (173), SKLB023 (174), and LY3200882 (175), are effective in treating HBV-HCC. The therapeutic vaccine Belagenpumatucel-L (Lucanix) (176) also exhibits efficacy in this setting. Studies show significant activation of the TGF-β pathway in immunotherapy-resistant tumors, with TGF-β often implicated in establishing suppressive tumor microenvironments (177, 178). The TGF-β and PD-1 pathways operate through independent, yet complementary, immunosuppressive mechanisms, enhancing cancer immune evasion (179, 180). Consequently, combining TGF-β inhibitors with PD-1 monoclonal antibodies presents a promising treatment approach for HBV- HCC.

## 4 Interaction of HBx and TGF-β in HCC

As described previously, HBx and TGF-β play distinct functions in HBV-HCC progression. In fact, HBx was shown to induce TGF-β expression early in HBV infection (25). Meanwhile, TGF-β can increase signaling pathway proteins in HBx pathogenesis (181), suggesting that TGF-β and HBx co-regulate specific signaling pathways that promote HCC. Additionally, regulating multiple pathways and epigenetic and genetic events by HBx mediates TGF-β’s participation in distinct ways in HBV-HCC progress pathogenetic mechanism.

### 4.1 HBx shifts TGF-β action from tumor suppression to tumorigenesis

As mentioned, the TGF-β pathway is involved in tumor suppression during early-stage tumorigenesis and tumor promotion in advanced cancers (94, 182–184). This dichotomous effect is determined by the phosphorylation status of the Smad3 protein, particularly its c-terminus or linker region (185). The linker region of Smad2/3 contains numerous conserved motifs subject to regulatory factors and post-translational modifications, such as phosphorylation (186, 187). The linker domain is phosphorylated at specific serine/threonine residues by cytoplasmic MAPKs and nuclear CDKs (20, 35–38). The phosphorylation of Smad2/3 in the linker region generates three types of phospho-isoforms: C-terminally phosphorylated Smad2/3 (pSmad2C/3C), linker-phosphorylated Smad2/3 (pSmad2L/3L), and dually phosphorylated Smad2/3 (pSmad2L/C and 3L/C) (188).

In normal epithelial homeostasis, TGF-β mediates pSmad3C signaling, inhibiting cell proliferation by interfering with cell cycle progression (189–191). This is accomplished via activating CDK inhibitors, such as p15INK4B and p21CIP1, and inhibiting c-Myc gene expression and cell cycle-related molecules (192–194). The pSmad3C pathway protects against cancer development, leading to transient Ras activation followed by growth inhibition and apoptosis. Furthermore, pSmad3C can regulate apoptosis-related protein expressions, including Bcl2 (195)^(p2)^.

Cytoplasmic Ras-related kinase activation during carcinogenesis, including MAPKs, transforms Smad3 signaling from an antitumor pSmad3C state to the oncogenic pSmad3L and pSmad2L/C pathways. JNK, a serine/threonine kinase activated by Ras, plays a crucial role in this conversion by defeating TβRI/pSmad3C-mediated growth arrest (196, 197). Smad3 is phosphorylated at Ser-213 upon activation of JNK (198), a site where RTK pro-inflammatory cytokines, growth factors, and, to a lesser extent, TGF-β can increase phosphorylation levels. It has been demonstrated that c-Myc overexpression can inhibit the Smad3-dependent transcription of p15INK4B and p21WAF1 proteins, thereby opposing cell cycle arrest (199). The JNK/c-Myc mitotic pathway inhibits the TRI/pSmad3C/P21WAF1-mediated growth arrest (200). Ser-213 phosphorylation of Smad3L via TβRI perturbs COOH-tail phosphorylation (26, 198, 201), promoting nuclear translocation and accelerating cell proliferation signals mediated by pSmadL (198). JNK-activated pSmad3L-mediated cell proliferation signal and TβRI-activated pSmad3C-mediated cell cycle arrest signal are mutually antagonistic. Mutations in essential pathway components can cause persistent Smad3 linker phosphorylation, so highly phosphorylated Smad3L likely reduces pSmad3C’s sensitivity to growth inhibition in tumor cells (26, 202–204). The overexpression of receptors by cancer cells modifies Smad3 phosphorylation (205). The Ras/JNK pathway controls both pSmad3C and pSmad3L. Strong Smad2L/C and Smad3L/C phosphorylation is observed in colorectal cancer EMT-related tumors (206). CDK4 converts TGF-β signal-mediated pSmad2/3C to malignant pSmad2L/C and 3L/C pathways (207). The interaction between pSmad2L/C and pSmad3L induces fibrogenic signals and liver fibrosis via PAI-1 (208). Increased PAI-1 transcription and ECM synthesis positively modulate liver fibrosis in hepatocytes (209).

**Figure 5:**
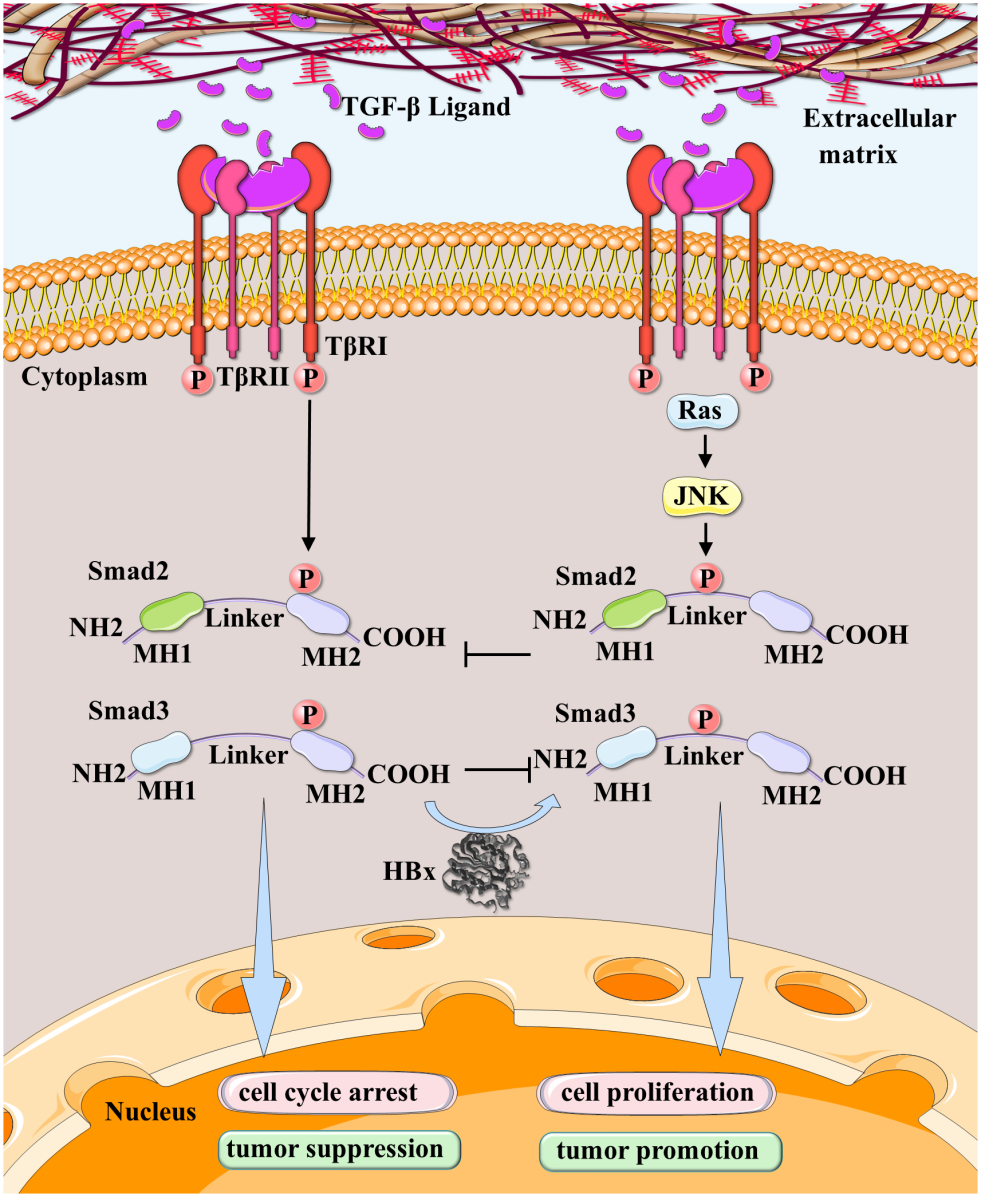
HBx participated in the conversion of dichotomous effects on HCC in the TGF-β pathway. JNK-activated pSmad2/3L-mediated cell proliferation signal and TβRI-activated pSmad2/3C-mediated cell cycle arrest signal are mutually antagonistic. HBx leads TGF-β signaling in hepatocytes to shift from the TβRI-dependent pSmad3C tumor-suppressive pathway to the JNK-dependent pSmad3L oncogenic pathway during carcinogenic stages.

It has been demonstrated that HBx overexpression induces the development of hepatic tumors by stimulating DNA synthesis (210). Studies have revealed that HBx shifts TGF-β signaling from the TβRI-dependent pSmad3C tumor-suppressive pathway to the JNK-dependent pSmad3L oncogenic pathway during carcinogenesis, as observed in biopsy samples from chronically HBV-infected patients and HBx transgenic mice with liver lesions (26). The proto-oncogene c-Myc, a target of TGF-β/SMAD signaling (211, 212), is involved in HCC malignant progression (213). pSmad3L/Smad4 triggers c-Myc transcription, whereas pSmad3C/Smad4 inhibits it. The two complexes antagonize each other and govern c-Myc expression. HBx’s presence may cause the signal to upregulate c-Myc and promote cancer cell growth (214) (**Figure 5**).

In conclusion, the interaction between HBx and TGF-β plays an essential role in hepatocarcinogenesis at various stages of HBV infection. TGF- β signaling has a dual function, promoting tumor suppression in the early stages of tumorigenesis while promoting tumor growth in advanced malignancies. Smad3’s function is determined by its phosphorylation status, with C-terminally phosphorylated Smad3 (pSmad3C) functioning as a tumor suppressor and linker-phosphorylated Smad3 (pSmad3L) contributing to oncogenesis. HBx modifies the TGF-β signaling pathway, redirecting it to the oncogenic pSmad3L pathway. This perturbation promotes the progression of the cell cycle, inhibits apoptosis, and accelerates the development of hepatocellular carcinoma.

### 4.2 Other HBx effects on TGF-β signaling

In prior studies, researchers revealed a correlation between HBx and TGF-β expression in HBV-infected cells. TGF-β1 level correlates positively with HBx protein expression in early-stage HBV infection, suggesting that HBx may directly or indirectly promote TGF-β1 expression. HBx forms a complex with Egr-1 protein and transactivates the TGF-β1 promoter via the Egr-1 binding site (25). HBx overexpression during HBV infection correlates with the release of secreted factors, particularly TGF-β, from HBx-transfected HCC cells or adjacent endothelial cells through increased CD133 expression, which induces invasion of these cells by EMT (210).

TGF-β1 inhibited cell proliferation and invasion in a study involving trophoblast cells (HTR-8/SVneo). Nevertheless, HBx activated the Smad pathway in HBx-transfected cells, resulting in the downregulation of E-cadherin and the upregulation of vimentin and N-cadherin, which reduced the apoptotic capacity and increased the invasive capacity of HTR-8/SVneo cells. The mechanism by which HBx shifts TGF-β signaling in HTR-8/SVneo cells needs additional investigation (211). Additionally, HBx inhibits the expression of the E-cadherin gene (CDH1) by activating TGF-β, which may be an additional mechanism for its downregulation of E-cadherin and promotion of tumor metastasis (212). The HTR-8/SVneo cell line is derived from cells that were grown from early human placental chorionic villi explants and transfected with a gene encoding the Simian Virus 40 large T antigen. Since it is not sourced from a liver cancer cell line, further foundational experiments are necessary to determine whether it can represent the general mechanisms within HBV-infected and liver cancer patients. This includes using human-derived liver cancer cell lines and replicating the studies in mouse models that simulate liver cancer to validate the results.

Epigenetic and genetic events affect the interaction between HBx and TGF-β. HBx can increase TGF-β expression via autophagy induction, and increased TGF-β upregulates lncRNA-ATB, thereby enhancing liver cancer cell migration and invasion (215). Both HBx and TGF-β1 stimulation induces significant overexpression of miR-199a-3p in hepatic progenitor cells (HPCs), thereby promoting HPCs oncogenic transformation via a JNK/c-Jun/miR-199a-3p-dependent pathway (216). C-terminal truncated mutants (ctHBx) commonly found in HCC tissue samples (217) decrease bone activin membrane-bound inhibitor (BAMBI) more than HBx alone. ctHBx significantly inhibits BAMBI promoter activity in the absence of the Wnt/β-catenin pathway, thereby reducing the inhibition of TGF-β1 and β-catenin and promoting malignancy (218).

In an HBx transgenic mouse model undergoing partial hepatectomy, TGF-β, Smad2, and phosphorylated Smad3/4 (ser423/425) were significantly overexpressed in the HBx transgenic mice liver compared to non-transgenic mice, indicating the impact of HBx on the TGF-β/Smad pathway, which promotes the progression of HCC (213). Furthermore, HBx disrupts the negative feedback loop between TGF-β and protein phosphatase magnesium-dependent 1A (PPM1A) by enhancing PPM1A ubiquitination and degradation, resulting in TGF-β pathway overactivation, HCC migration, and invasion (219). HBx also stabilizes the binding of the Smad complex to the transcriptional machinery and facilitates the nuclear transposition of Smads, thereby amplifying TGF-β signaling (220).

Contrary to previous findings, some studies suggest a negative correlation between HBx and TGF-β activation. For example, HBx inhibits TGF-β-induced apoptosis by linking Src to PI3K, thereby activating the PI3K/Akt signaling pathway (221, 222). And another study showed that cells transfected with HBx exhibited reduced expression of the TGF-β type II receptor, resulting in a weaker TGF-β1 response and reduced growth inhibition in response to TGF-β1 (214). Furthermore, research also shows that HBx plays different roles at hepatocyte cell line. In normal liver cells, HBx induces cell cycle arrest by elevating TGF-β and p27 levels, leading to G1 or G2 phase blocks that facilitate HBV replication. In HBV-infected HCC cells, however, HBx may accelerate carcinogenesis by promoting cell cycle progression through the downregulation of TGF-β and the p53/27/21 pathway (223). Despite these findings, the overall trend indicating tumor progression via the HBx/TGF-β axis aligns with earlier results. This may be attributed to the paradoxical roles of TGF-β at different stages of tumor progression or variations in experimental conditions.

**Figure 6:**
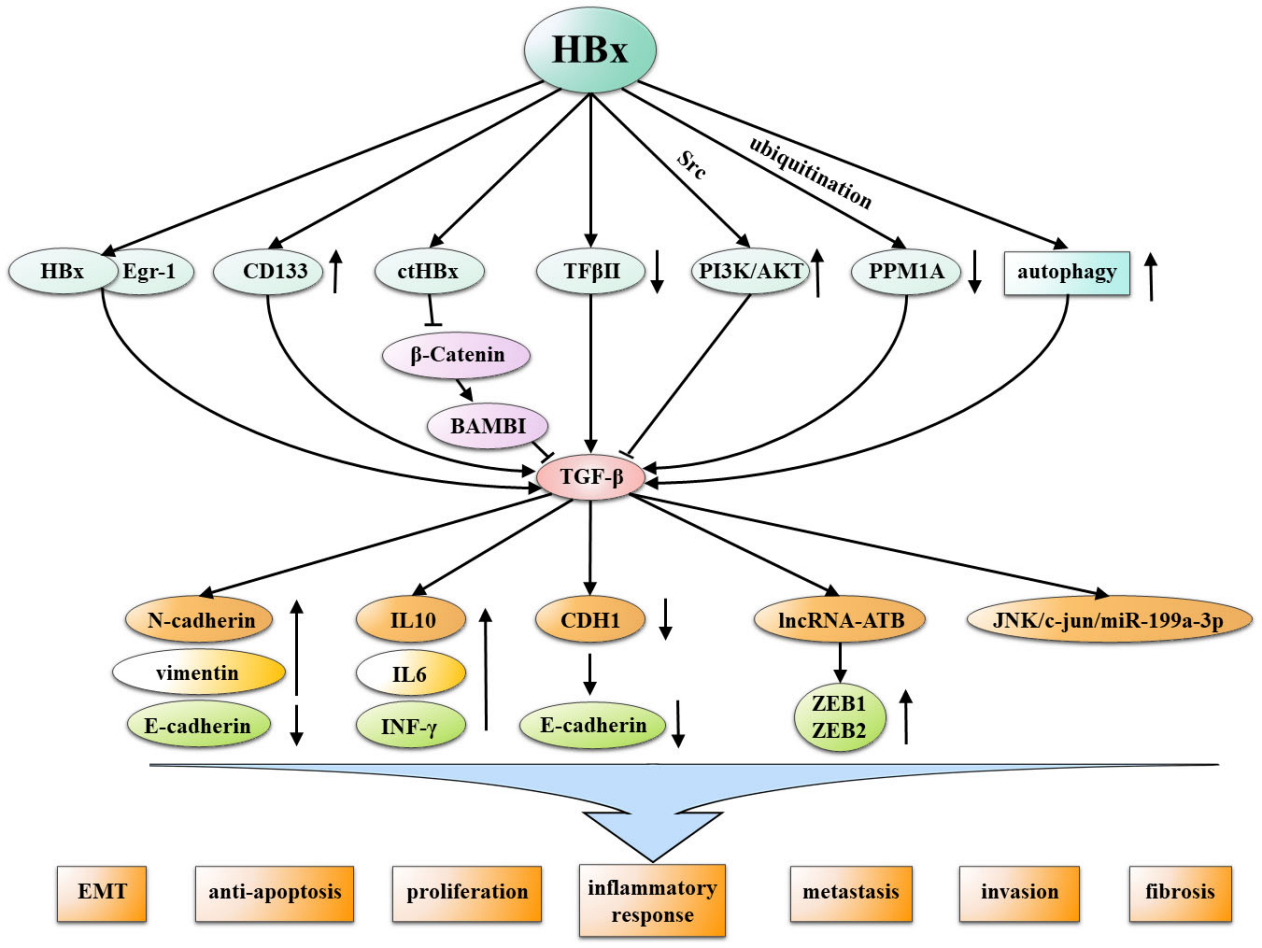
Multiple mechanisms by which HBx interacts with TGF-β in the non-SAMD pathways to promote HCC. HBx and TGF-β mutually interact through various mechanisms, inducing malignant characteristics in development of HCC, including EMT, anti-apoptosis, proliferation, inflammatory responses, metastasis, invasion, and fibrosis.

It is evident that the TGF-β signaling pathway is primarily influenced and regulated by HBx, particularly within HBV-infected cells. It is worth noting that HBx has been shown to lead to malignant transformation of HCC by influencing dysregulation of TGFB and thereby activating multiple cancer-promoting mechanisms. including EMT, anti-apoptosis, proliferation, inflammatory responses, metastasis, invasion, and fibrosis. Therefore, researchers need to pay more attention to the interaction mechanism of these HBx-TGF-β axis, and develop anti-tumor drugs that can target the common pathway of this axis, bringing new hope to the drug treatment of hepatocellular carcinoma. (**Figure 6**).

## 5 Conclusion and discussion

The incidence and mortality rates of liver cancer have garnered widespread attention within the academic community. With the advancement of liver cancer research, there has been a deeper understanding and significant progress in elucidating carcinogenic molecular mechanisms. Various mechanisms, such as DNA damage, immune evasion, epigenetic alterations, and genomic mutations, have been emphasized in studies and are considered crucial in promoting HCC through HBx. Notably, as a key signaling pathway, TGF-β plays dual roles in HCC, transitioning from an early anticancer effect to a late pro-cancer effect. HCC can acquire invasive tumor characteristics such as EMT and aberrant proliferation through this pathway. In numerous recent studies, it has been demonstrated that HBx and TGF-β signals interfere and interact with one another in HCC and collectively regulate HCC progression. With future technological advances, it is believed that more in-depth studies will be necessary to reveal the HBx oncogenic mechanism, a star protein, and TGF-β, a potential HCC therapeutic target, and to design effective clinical management strategies for HBV-correlated HCC patients. In the near future, these efforts will provide HCC patients with more effective and sensible options for receiving targeted therapies.

## Author contributions

WY: Writing – review & editing, Writing – original draft. DR: Writing – review & editing, Writing – original draft. FF: Writing – review & editing, Writing – original draft. HL: Writing – review & editing, Writing – original draft. ZZ: Writing – review & editing, Writing – original draft. HD: Writing – review & editing, Writing – original draft.

## Glossary

**Table:**

HCC	hepatocellular carcinoma
HBV	Hepatitis B virus
HBx	Hepatitis B virus X protein
cccDNA	covalently closed circular DNA
TGF-β	transforming growth factor-β
NAFLD	nonalcoholic fatty liver disease
rcDNA	relaxed circular DNA
ORFs	overlapping open reading frames
aa	amino acids
TFs	transcription factors
EMT	epithelial-mesenchymal Transition
ASPH	Aspartate β-hydroxylase
TFIIH	Transcription factor IIH
HR	homologous recombination
DSBs	DNA double-strand breaks
TLR3	Toll-like receptor 3
PRR	pattern recognition receptor
TRIF	TIR-domain-containing adaptor-inducing interferon-β
IFN	interferon
IPS-1	IFN-β promoter stimulator 1
ADAR1	adenosine deaminases acting on RNA 1
SOCS-1	suppresses cytokine signaling-1
ATRA	all-trans retinoic acid
PTPN13	phosphatase nonreceptor type 13
IGF2BP1	insulin-like growth factor 2 mRNA-binding protein 1
WDR5	WD repeat domain 5 protein
H3	histone 3
H3K4me3	H3 lysine 4-trimethylation
H3K9me	histone 3 lysine 9 ethylation
HP1	heterochromatin protein-1 factor
SETDB1	SET domain bifurcated1
SIRT2	Sirtuin 2
miRNAs	microRNAs
PDCD4	programmed cell death 4
HBx-LINE1	HBX-LINE1-674
NUSAP1	nucleolar spindle-associated protein 1
HPIP	hematopoietic pre-B cell leukemia TF-interacting protein
lncRNA	long non-coding RNA
PRMT1	type I protein arginine methyltransferase
ZHX2	Zinc fingers and homeoboxes 2
TRERNA1	translation regulatory lncRNA 1
NRAS	neuroblastoma RAS
MAPK	Mitogen-Activated Protein Kinase
Erα	estrogen receptor
APC	adenomatous colon polyps
PPI	protein-protein interactions
ROSs	reactive oxygen species
Ct-HBx	C-terminus truncated X protein
NFATC2	nuclear factor of activated T cells 2
MMP10	matrix metalloproteinase 10
Cav1	Caveolin-1
LRP6	lipoprotein receptor-related protein 6
FRMD5	FERM domain containing 5
HBX-C30	30 aa deletion from HBx C terminus
FXR	farnesoid X receptor
F30V	a specific HBx genetic mutation
LAP	latency-associated peptide
LTBP	latent TGF-β binding protein
ECM	extracellular matrix
Erk1/2	extracellular signal-regulated kinases1/2
PI3K	phosphatidylinositol-3-kinase
JNK	c-Jun amino-terminal kinase
CDKs	cyclin-dependent kinases
Nox4	niacinamide adenine dinucleotide phosphate oxidase-4
RB	retinoblastoma protein
hTERT	human telomerase reverse transcriptase
DAPK	death-associated protein kinase
ER	endoplasmic reticulum
Bcl-2	B-cell lymphoma 2
TNF	tumor necrosis factor
TRAIL	TNF-associated apoptosis-inducing ligand
EGFR	epidermal growth factor receptor
TAMs	Tim-3 transcription in tumor-associated macrophages
SCF	stem cell factor
SIP1	Smad interacting protein-1
H3K9	histone H3 lysine9
α-SMA	α-smooth muscle actin
FSP-1	fibroblast specific protein1
MDSCs	myeloid-derived suppressor cells
HSCs	hepatic stellate cells
CAFs	tumor-associated fibroblasts
Treg	regulatory T cells
CTLS	cytotoxic T lymphocytes
PD-L1	programmed death ligand 1
TCR	transmission of T cell antigen receptor
PTMs	post-translational modifications
SP	SerPro
TP	ThrPro
Abs	antibodies
PAI-1	plasminogen activator inhibitor-1
CDH1	E-cadherin gene
PPM1A	protein phosphatase magnesium-dependent 1A
lncRNA-ATB	lncRNA activation by TGF-β
HPCs	hepatic progenitor cells
BAMBI	bone activin membrane-bound inhibitor
